# The identification of a CD47-blocking “hotspot” and design of a CD47/PD-L1 dual-specific antibody with limited hemagglutination

**DOI:** 10.1038/s41392-020-0121-2

**Published:** 2020-03-06

**Authors:** Rui Shi, Yan Chai, Xiaomin Duan, Xiaoshan Bi, Qingrui Huang, Qihui Wang, Shuguang Tan, George F. Gao, Jianhua Zhu, Jinghua Yan

**Affiliations:** 1 CAS Key Laboratory of Microbial Physiological and Metabolic Engineering, Institute of Microbiology, Chinese Academy of Sciences, 100101 Beijing, China; 2 University of Chinese Academy of Sciences, 100049 Beijing, China; 3 CAS Key Laboratory of Pathogenic Microbiology and Immunology, Institute of Microbiology, Chinese Academy of Sciences, 100101 Beijing, China; 4 Institute of Physical Science and Information Technology, Anhui University, 230601 Hefei, China; 5 Department of Oncology, Fourth Medical Center of PLA General Hospital, 100048 Beijing, China

**Keywords:** Drug development, Structural biology

Dear Editor,

By targeting the programmed cell death 1 (PD-1) pathway with monoclonal antibodies (mAbs), immune checkpoint therapy (ICT) has achieved unprecedented clinical success in the treatment of multiple tumors.^1,2^ Cancer cells evade the host immune system via both the tolerance of T cells and functional suppression of innate immune cells.^3^ CD47 provides a “do not eat me” signal by binding to signal regulatory protein alpha (SIRPα) to prevent innate immune cells from attacking host cells.^4^ Recently, macrophages were found to restore antitumor reactivity by blocking the interaction between upregulated CD47 on tumor cells and SIRPα on innate immune cells.^5^ However, it remains unknown whether there are blocking “hotspots” on CD47 for mAb-based anti-CD47 therapy or additional blocking hotspot regions within CD47 for therapeutic mAb development. Although it is overexpressed on tumor cells, CD47 is also expressed in many normal cells, including red blood cells and platelets.^6^ Some validated CD47-blocking mAbs under clinical investigation induce hemagglutination and anemia.^7^ Thus, designing an engineered CD47-blocking antibody to exert a therapeutic effect with limited hemagglutination is needed.

To overcome this obstacle, we identified a CD47-targeting mAb and evaluated its enhanced macrophage-mediated phagocytosis capacity and tumor suppression efficacy. We screened a panel of 50 murine mAbs from mice immunized with human CD47 and identified an efficient CD47/SIRPα-blocking mAb, m4C1 (Fig. S1a). m4C1 was subsequently humanized based on sequence homology and computational prediction (Fig. S1c). Humanized 4C1 (h4C1) blocked the CD47/SIRPα interaction as efficiently as m4C1 (Fig. S1e). Surface plasmon resonance (SPR)-binding assays demonstrated that h4C1 bound to the human CD47-ECD antigen with a *K*_D_ of 0.85 nM (Fig. S1d), which is comparable to that of m4C1 (Fig. S1b, Table S1). To investigate the macrophage-mediated phagocytosis activity of h4C1, a tumor cell phagocytosis assay was conducted with bone marrow-derived macrophages (BMDMs). We found that h4C1 exhibited significant phagocytosis in Raji cells, with an EC_50_ of 7.30 ng/mL (Fig. 1a).

**Fig. 1:**
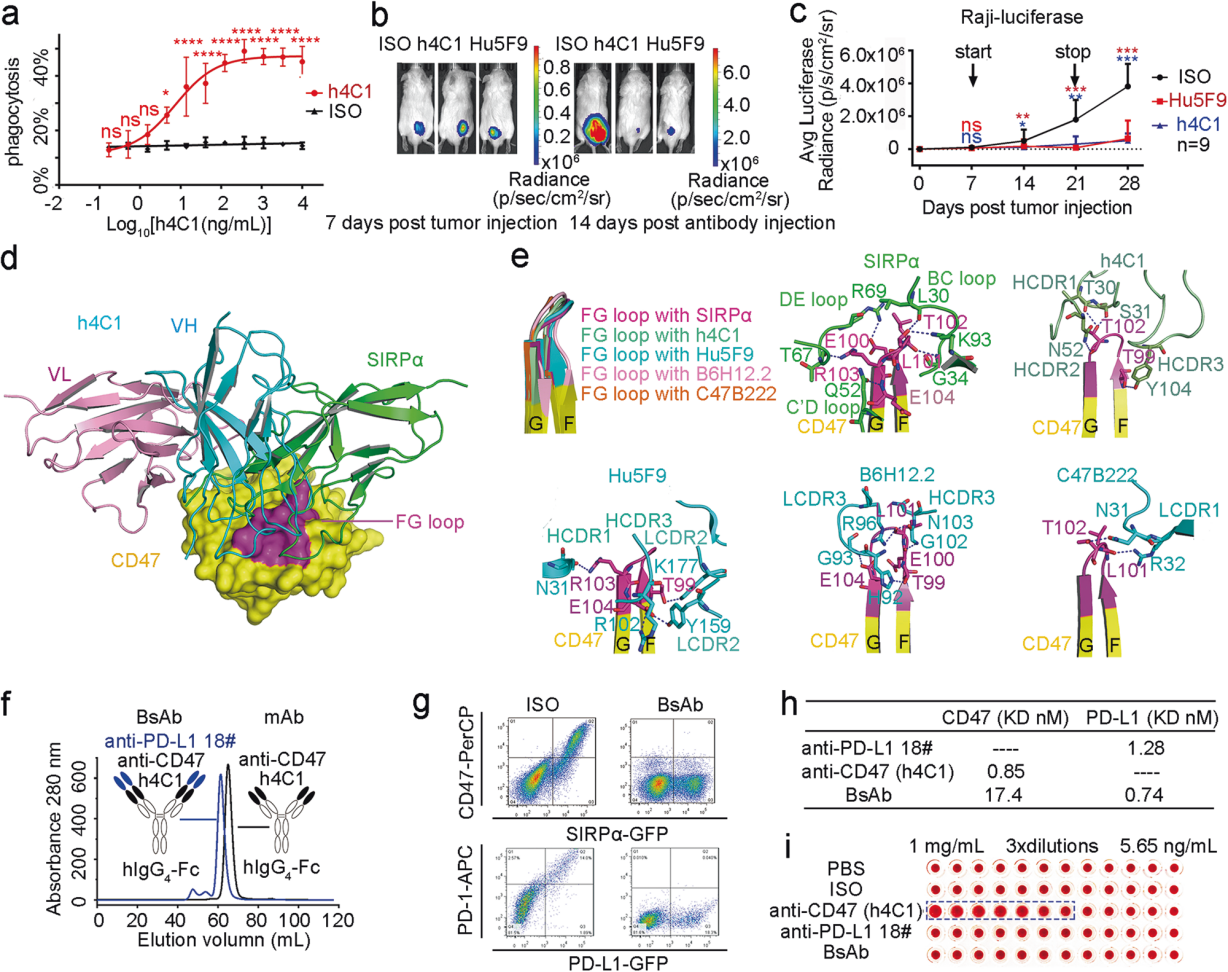
The FG loop of CD47 as a blocking hotspot, and the construction of a CD47/PD-L1 dual-targeting antibody with no hemagglutination. **a** The EC_50_ (7.30 ng/mL) of h4C1 was calculated by fitting the phagocytosis index of Raji cells from a serially diluted antibody to a sigmoidal dose–response curve. Bars represent the mean ± SD, and the statistical significance was analyzed using two-way ANOVA with Sidak correction for multiple comparisons; ns, not significant, *p < 0.05 and ****p < 0.0001. The data presented here are representative of three independent experimental results. **b** Three groups of mice were enrolled with nine mice in each group. Treatment with an isotype IgG was used as a negative control, and Hu5F9 was used as a positive control. Representative bioluminescence images of Raji tumors at Days 7 and 28 post-tumor inoculation are presented. **c** Comparative analysis of average luciferase radiances in each treatment group (*n* = 9). The data are presented as the mean ± SD. *p-*values were calculated using Student’s *t*-test compared to the isotype IgG treatment group (*p < 0.05, **p < 0.01, and ***p < 0.001). **d** Superimposition of the h4C1/CD47 complex and SIRPα/CD47 (PDB ID: 2JJS) reveals the stereospecific competition between h4C1 and SIRPα. The CD47-ECD/h4C1 structure superimposed on CD47-ECD/SIRPα demonstrates stereo-specific hindrance. CD47-ECD is shown as a cartoon with a translucent surface (yellow). The FG loop on the CD47-ECD surface is in purple. **e** Comparison of the FG loop of the CD47-ECDs from the complex structures. Binding details of the FG loop of CD47 with SIRPα. Residues interacting between the FG loop on CD47-ECD and the antibody h4C1, Hu5F9, B6H12.2, and C47B222. CD47-ECD is shown as a cartoon in yellow, and the FG loop is in purple for emphasis. h4C1 is displayed as a cartoon in olive green; all other blocking agents are also in olive green or cyan. Residues involved in hydrogen bond interactions are shown as sticks, and the hydrogen bonds are shown as dashed blue lines. **f** Schematic of the CD47/PD-L1 dual-targeting BsAb. h4C1 (black) is linked with the variable domains from anti-PD-L1 #18 (blue). Constant regions are the human IgG_4_ isotype (white). **g** The CD47/PD-L1 dual-targeting antibody can block both the CD47/SIPRα and PD-1/PD-L1 interactions in a flow cytometry-based assay. SIRPα or PD-1 was transiently expressed on the HEK 293T cell surface with GFP, and SIPRα-expressing or PD-1-expressing HEK 293T cells were stained with CD47-ECD or PD-L1-ECD proteins, which were preincubated with an isotype IgG or a dual-targeting antibody. **h** Binding characteristics of the CD47/PD-L1 dual-targeting antibody. Antibodies were immobilized on the chip, while serial dilutions of the antigen were then flowed over the surface of the chip. The binding affinities (*K*_D_) are labeled accordingly. **i** The CD47/PD-L1 dual-targeting antibody with limited hemagglutination. Antibodies were triple gradient dilutions from 2 mg/mL to 11.3 ng/mL. Then, equal volumes of antibodies and human RBCs were mixed. Hemagglutination was defined as red or brown flocculation in the supernatant. Compared to h4C1, the CD47/PD-L1 dual-targeting antibody displays no hemagglutination.

We further assessed the in vivo tumor suppression efficacy of the CD47-targeting antibody, h4C1, in a mouse model. We subcutaneously engrafted luciferase-labeled Raji cells into the backs of immune-compromised NCG mice. Seven days after the engraftment, the mice were administered h4C1, Hu5F9 (a validated CD47-blocking mAb under clinical investigation^7^), or isotype control mAb daily for 2 weeks (Fig. S2a). The average luciferase intensity in the h4C1-treated group was significantly lower than that of the isotype mAb-treated group (Figs. 1b, c and S2b–d). Treatment with h4C1 also resulted in a substantially improved survival of the tumor-bearing mice (Fig. S2e). Taken together, these results suggest that h4C1 represents a potential therapeutic mAb for lymphoma.

The binding characteristics of h4C1 to CD47 were subsequently explored through the determination of the h4C1/CD47 complex structure (Fig. S3a, b, Table S2). The overall structure reveals that h4C1 binds to CD47 with a buried surface area of 688 Å^2^. All three CDRs of heavy chain (VH) and LCDR2 provide contacts with CD47-ECD, while LCDR1 and LCDR3 are not engaged (Fig. S4). The findings indicate that h4C1 exhibits a VH-dominated interaction with CD47, while the binding of h4C1 is mainly located on three loops of CD47 (FG, BC, and C’C”).

To analyze the mechanism of CD47 antagonism by h4C1, the structure of the h4C1-Fab/CD47-ECD complex was superimposed onto the CD47-ECD/SIRPα complex (PDB ID: 2JJS). Detailed comparison of reciprocal binding areas between the two complexes revealed multiple striking parallels. Specifically, the FG loop of CD47-ECD inserts into a groove on the surface of domain I of SIRPα and contributes more than half of the interfacial surface.^4^ Similarly, the FG loop of CD47-ECD dominates the interaction with VH of h4C1. The binding of h4C1 to CD47 displayed substantial stereospecific hindrance to the binding of h4C1 to SIPRα (Fig. 1d).

There are five potential N-linked glycosylation sites on CD47-ECD. In the complex structure of h4C1/CD47, glycosylation modifications were observed at all five of these sites in CD47 (Fig. S5a). Notably, the glycan chain on N32 substantially stretches toward the h4C1/CD47 interface and contacts h4C1 (Table S3 and Fig. S3c). Additionally, glycosylated N55 is also near the h4C1/CD47 interface. The binding profiles of h4C1 to N32A-mutated or N55A-mutated CD47 proteins were further analyzed with SPR to investigate the glycosylation dependency of the h4C1/CD47 interaction. SDS–PAGE analysis revealed that N32A-mutated or N55A-mutated CD47 proteins with a reduction in molecular weight indicates the existence of the N-glycosylation modification at these sites (Fig. S3d). The SPR assays demonstrated that no substantial differences were observed in the binding affinity of WT CD47-ECD (Fig. S1d) and N32A-mutated or N55A-mutated CD47-ECD (Fig. S5b, c). Therefore, the binding of h4C1 to CD47 is not affected by glycosylation modifications of CD47.

Several reported complex structures of CD47-blocking agents enabled us to compare these binding characteristics and to elucidate sites for preferential blocking. Overall, these mAbs mainly bind to the FG loop of CD47, and the conformation of the FG loop displayed no substantial differences, indicating the structural conservation of the loop (Fig. 1e). In particular, the FG loop of CD47 dominates the binding to SIPRα and to all of the mAbs. The binding of Hu5F9 to the FG loop of CD47 is mainly mediated by HCDR1, HCDR3, LCDR1, and LCDR2. The complex structure of CD47 and a murine mAb that showed potential tumor suppression efficacy,^8^ B6H12.2, also exhibited FG loop-dominated binding to the mAb. The complex structure of C47B222/CD47 shows that the FG loop of CD47 also plays a critical role in binding to C47B222 (Fig. 1e). Therefore, the FG loop of CD47 dominates the interactions with these mAbs and could serve as a blocking “hotspot” for the design of next-generation anti-CD47 drugs in the future.

To reduce the hemagglutination of CD47-targeting mAb, we constructed a bispecific antibody (BsAb) in a dual-variable-domain immunoglobulin (DVD-Ig) format, and this antibody was expected to reduce the binding affinity to CD47. We introduced the variable domains from a validated PD-L1-targeting mAb #18 (CN Patent: 201810952740.3) onto h4C1 to enhance both innate and adaptive antitumor immune responses (Fig. 1f). A cell-based blocking assay showed that the BsAb maintained the blocking efficacy for both the CD47/SIRPα and PD-1/PD-L1 interactions (Fig. 1g). SPR was used to characterize the binding of the BsAb to PD-L1 or CD47, and a substantially reduced binding affinity was observed between BsAb and CD47; however, the binding affinity remained comparable to the binding of PD-L1 (Fig. 1h). Furthermore, a hemagglutination assay revealed that compared to h4C1 mAb, the CD47/PD-L1 BsAb exhibited substantially reduced hemagglutination in a wide range of concentrations in vitro (Fig. 1i). Therefore, the DVD-Ig CD47/PD-L1 BsAb design was able to maintain the blocking efficacy and reduce hemagglutination. Future investigations into the antitumor efficacy and hemagglutination should be conducted in a PD-L1-positive and CD47-positive mouse model.

In summary, the CD47-specific h4C1 mAb, which displayed substantial tumor suppression efficacy, could serve as a promising agent for future clinical investigations. Complex structure comparative analysis indicates that the FG loop of CD47 may serve as a blocking hotspot for the innate immune checkpoint. Notably, compared with a single anti-CD47 molecule, DVD-Ig that targets CD20 and CD47 with a reduced affinity receives the equivalent benefits of tumor treatment in vivo.^9^ The design of a CD47/PD-L1 dual-targeting BsAb with limited hemagglutination provides insight into next-generation anti-CD47 drugs to improve tumor ICT.

## Supplementary information

Supplemental Material

Supplemental Material
