# Supplementary material for: The identification of a CD47-blocking “hotspot” and design of a CD47/PD-L1 dual-specific antibody with limited hemagglutination
Source: Signal Transduct Target Ther. 2020 Mar 6;5:16. doi: 10.1038/s41392-020-0121-2 (PMC7058617; doi:10.1038/s41392-020-0121-2)
Supplement: Supplementary file 1 — Supplemental Material [file 41392_2020_121_MOESM1_ESM.docx]

**Supplemental Material for**

**Identification of a CD47 blocking “hotspot” and design of a CD47/PD-L1 dual-specific antibody with limited hemagglutination**

Rui Shi, Yan Chai, Xiaomin Duan, Xiaoshan Bi, Qingrui Huang, Qihui Wang, Shuguang Tan, George F Gao, Jianhua Zhu and Jinghua Yan

Contact: Jinghua Yan: yanjh@im.ac.cn and Jianhua Zhu: zjhua168@sohu.com

**Supplementary information, Figures and Tables**

Fig.S1 Identification and humanization of CD47 specific blocking antibody h4C1.

Fig.S2 h4C1 relieves lymphoma burden in xenograft mouse models.

Fig.S3 The complex protein of CD47-ECD/h4C1-Fab and N32A- or N55A-mutated CD47 protein.

Fig.S4 The crystal structure of the h4C1 and CD47-ECD complex.

Fig.S5 N-glycosylation of CD47-ECD and glycosylation-independent binding of h4C1.

Fig.S6 Binding characteristics of CD47/PD-L1 co-targeting BsAb.

Table S1. Binding characteristics of CD47 monoclonal antibodies.

Table S2. Crystallographic data collection and refinement statistics.

Table S3. Residues contributed interaction between h4C1 and CD47.

**Supplementary information, Materials and Methods**

**Supplementary information, References**


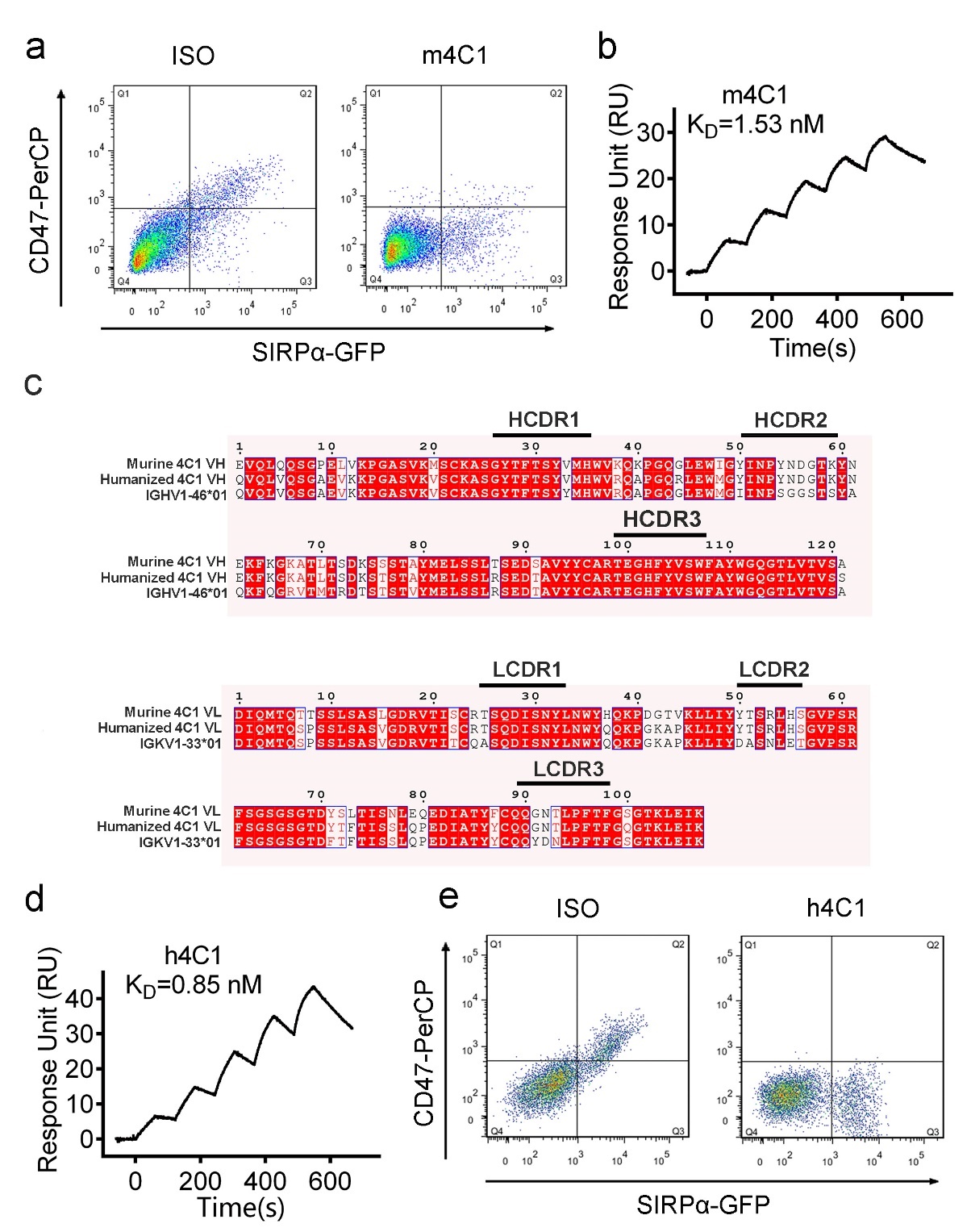
**Fig.S1 Identification and humanization of CD47 specific blocking antibody h4C1. (a)** The m4C1 mAb can block CD47 binding to SIPRα in flow cytometry-based assay. SIRPα was transiently expressed on the HEK 293T cell surface with EGFP, and the SIPRα-expressing HEK 293T cells were stained with CD47-ECD protein pre-incubated with isotype IgG or m4C1. **(b)** The binding affinity between m4C1 and CD47-ECD obtained from HEK 293T cells was determined using a single-cycle BIAcoreT100^®^ system. m4C1 was immobilized on the chip, while serial dilutions of CD47-ECD protein were then flowed over the chip surface. The binding affinity (K_D_) is labeled accordingly. **(c)** Sequence alignments highlighting the humanization strategy of m4C1 by retaining all of the CDRs and substituting the remaining amino acids for the corresponding residues in the human immunoglobulins. The human IGHV1-46*01, which exhibits the highest sequence identity to m4C1 in the heavy chain, was selected as the humanization backbone for the H chain, while IGKV1-33*01 was selected as the humanization backbone for the L chain. **(d)** The binding affinity between h4C1 and CD47-ECD obtained from HEK 293T cells was determined using a single-cycle BIAcoreT100^®^ system. The binding affinity (K_D_) is labeled accordingly. **(e)** The h4C1 mAb can block CD47 binding to SIPRα in flow cytometry-based assay. SIRPα was transiently expressed on the HEK 293T cell surface with GFP, and the SIPRα-expressing HEK 293T cells were stained with CD47-ECD protein pre-incubated with isotype IgG or h4C1.


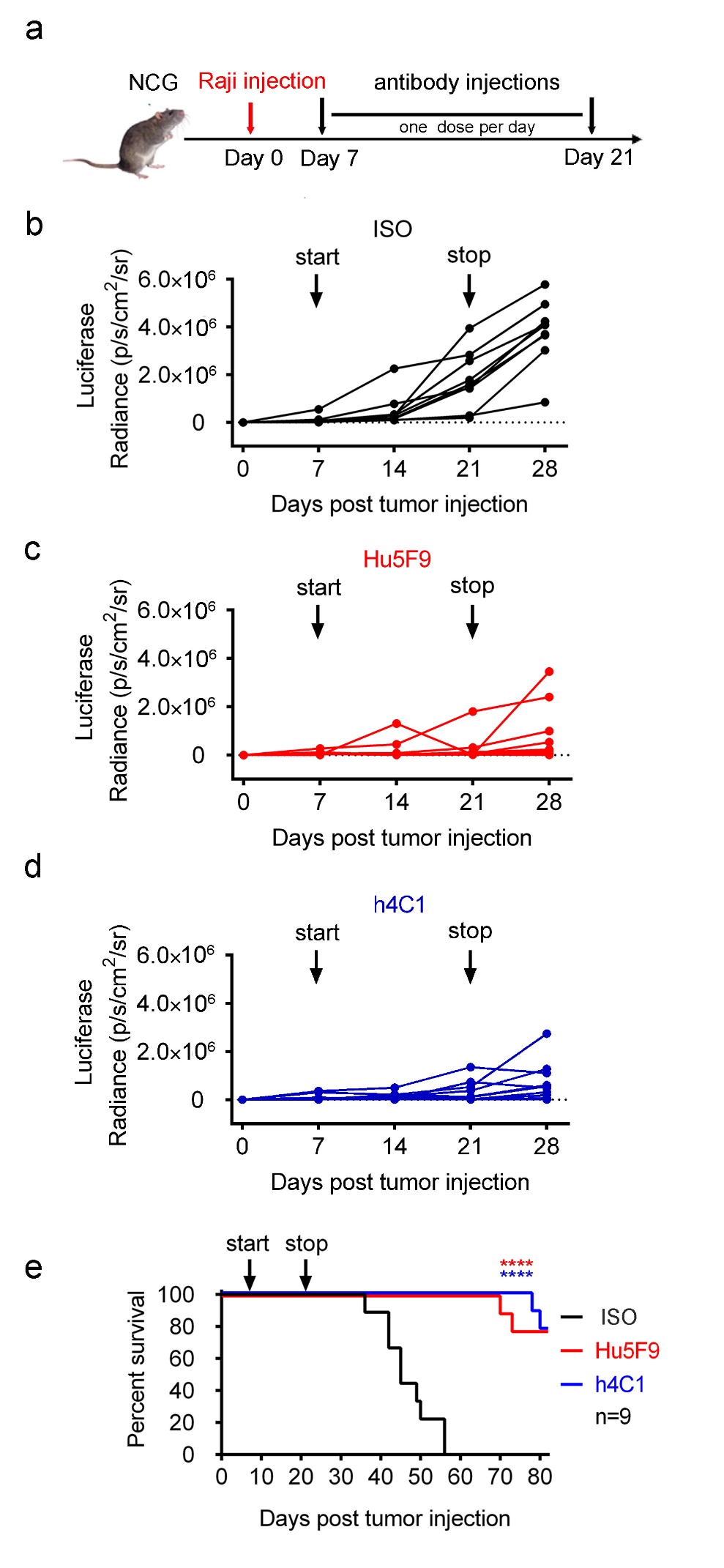


**Fig.S2 h4C1 relieves lymphoma burden in xenograft mouse models. (a)** Schematic diagram of the experimental workflow. NCG mice were inoculated with luciferase-labeled Raji cells. Seven days after Raji cell inoculation, mAbs were administrated (*i.p.*) every day for 14 days. The signal intensities of luciferase radiance from the tumor were monitored weekly after tumor inoculation. **(b-d)** Dynamic changes of the bioluminescence values for each mouse in the isotype IgG, Hu5F9, and h4C1 treatment groups after lymphoma inoculation. Arrows indicate the start (Day 7) and stop (Day 28) of the injections. **(e)** Survival of xenograft models bearing lymphoma treated with the indicated therapeutics. *p-*values were calculated using the Mantel-Cox test in comparison with that of the isotype IgG group (*****p* < 0.0001). The arrows indicate the start (Day 7) and end (Day 21) of the mAb administration.


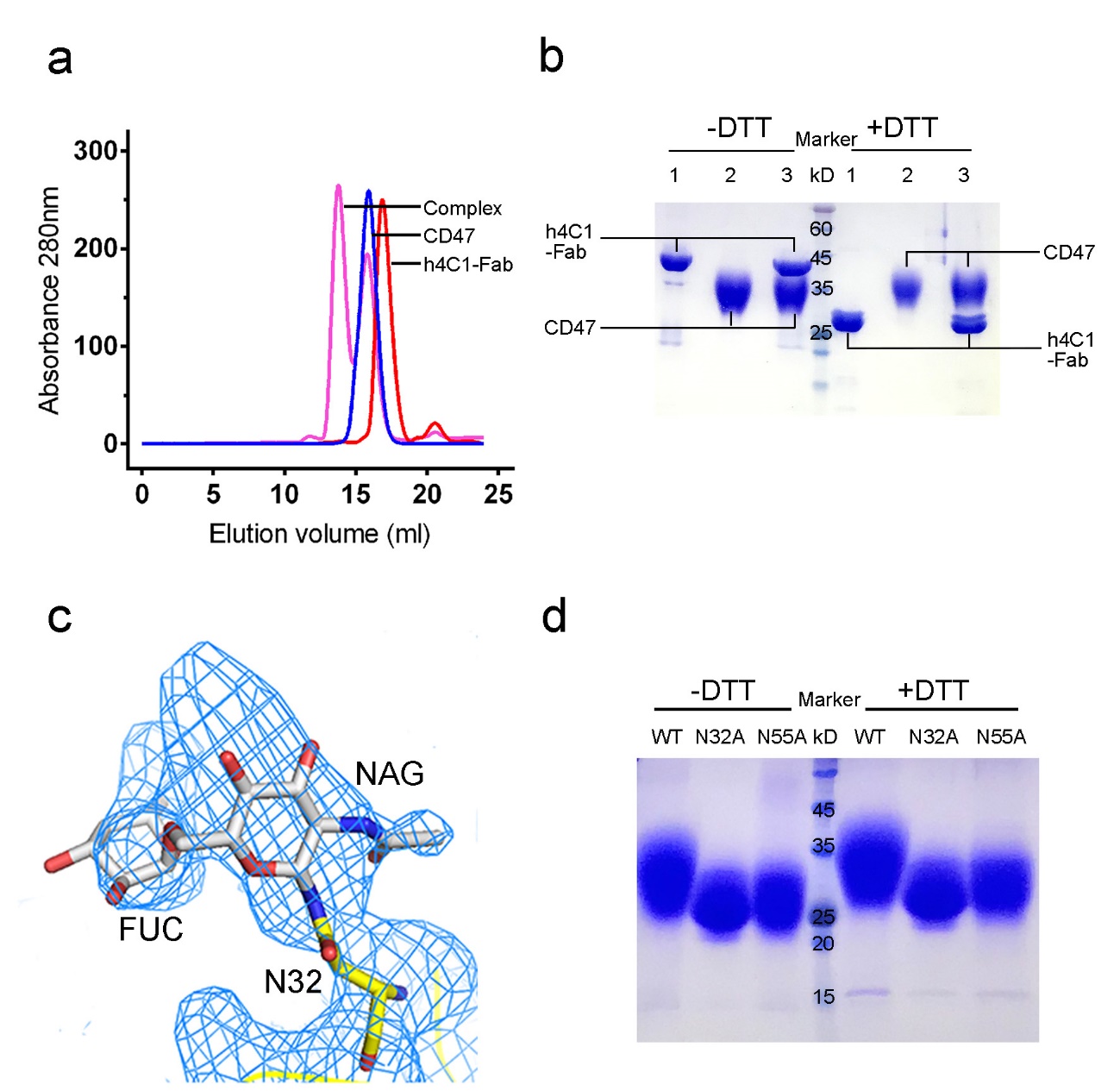


**Fig.S3 The complex protein of CD47-ECD/h4C1-Fab and N32A- or N55A-mutated CD47 protein. (a)** Gel filtration profiles of h4C1-Fab (red), CD47-ECD (blue), and the CD47-ECD/h4C1-Fab complex (pink) were analyzed by size-exclusion chromatography as indicated. **(b)** The SDS-PAGE analyses are shown in non-reducing (-DTT) or reducing (+DTT) conditions. **(c)** The electron density map of the N32 N-linked glycosylation is represented in yellow. Two glycans consisting of one NAG (white) and one FUC (white) can be observed. **(d)** SDS-PAGE analysis of the molecular reduction of N-glycosylation single site-mutated CD47-ECD protein. Compared to the WT CD47-ECD protein, the N32A or N55A N-glycosylation single site mutation induced a molecular weight reduction.
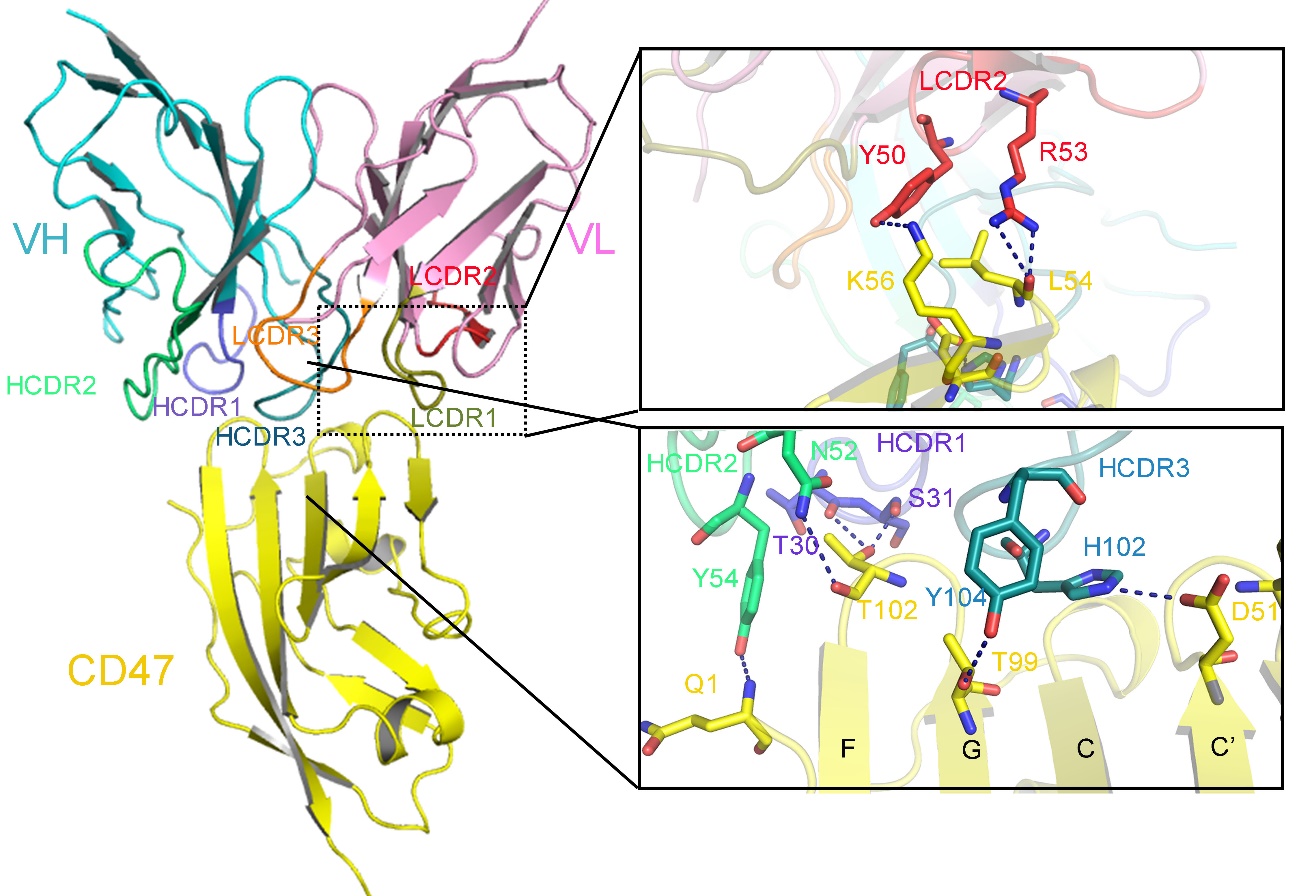


**Fig.S4** **The crystal structure of the h4C1 and CD47-ECD complex.** The variable fragment of h4C1 is shown as a cartoon with the HCDR1, HCDR2, and HCDR3 loops from the VH domain (cyan) colored in blue, light green, and deep teal, while the LCDR1, LCDR2, and LCDR3 loops from the VL domain (pink) are colored in deep olive, red, and orange, respectively. The binding details between h4C1 and CD47 are presented with amino acids from VL (upper right panel) and VH (lower right panel), which form hydrogen bond interactions with amino acids from CD47. The hydrogen bonds are shown as dashed blue lines.


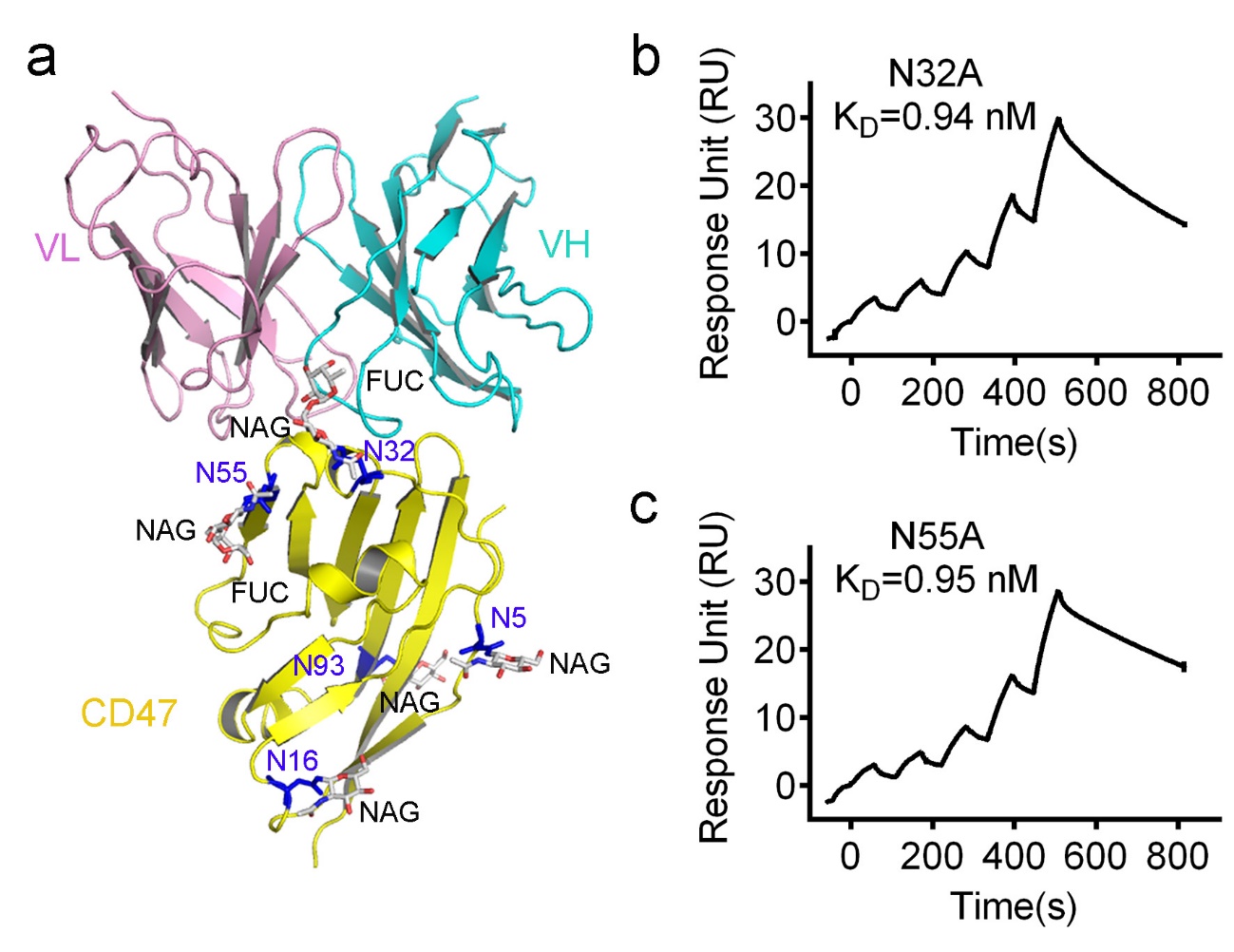


**Fig.S5 N-glycosylation of CD47-ECD and glycosylation-independent binding of h4C1. (a)** Complex structure of h4C1/CD47 with glycans depicted as sticks in gray. Five potential N-linked glycosylation sites (N5, N16, N32, N55, and N93) are shown as sticks in blue. **(b-c)** SPR assay characterization of the binding between h4C1 and N32A- or N55A-mutated CD47-ECD protein using a BIAcoreT100^®^ system. h4C1 was immobilized on the chip, while serial dilutions of mutant proteins were then flowed over the chip surface, respectively. The binding affinities (K_D_) are labeled accordingly.


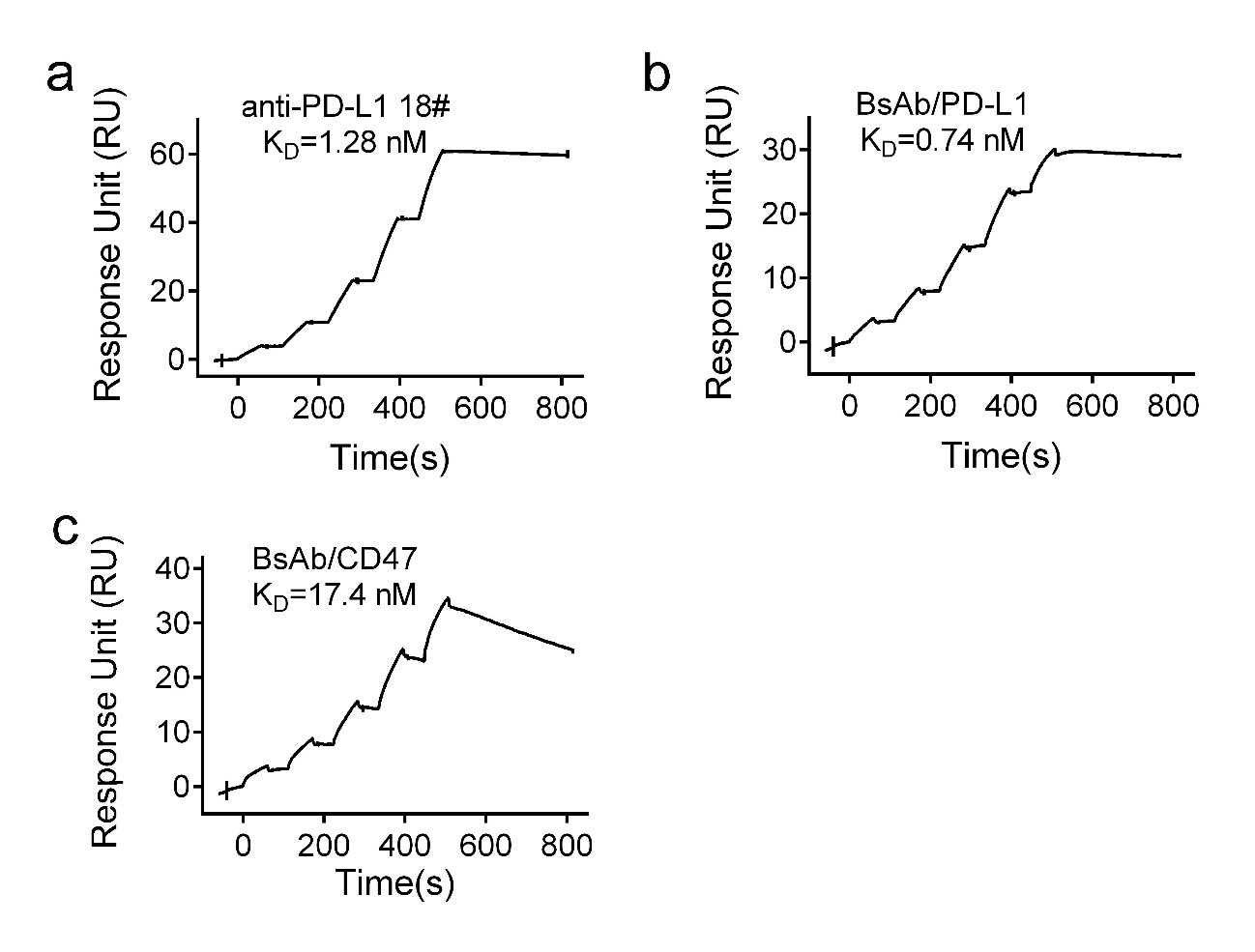
**Fig.S6 Binding characteristics of CD47/PD-L1 co-targeting BsAb. (a-b)** SPR assay characterization of the binding between anti-PD-L1 18# or BsAb and PD-L1-ECD protein using a BIAcoreT100^®^ system. **(c)** Binding assay between BsAb and CD47-ECD protein using a BIAcoreT100^®^ system. Antibodies were immobilized on the chip, while serial dilutions of mutant proteins were then flowed over the chip surface, respectively. The binding affinities (K_D_) are labeled accordingly.

**Table S1. Binding characteristics of CD47 monoclonal antibodies.**

| mAb | K_a_ (1/Ms) | K_d_ (1/s) | K_D_ (M) |
| --- | --- | --- | --- |
| m4C1 | 1.13×10^6^ | 1.72×10^-3^ | 1.53×10^-9^ |
| h4C1 | 3.16×10^6^ | 2.67×10^-3^ | 0.85×10^-9^ |

**Table S2. Crystallographic data collection and refinement statistics.**

|  | **h4C1-Fab/CD47** |
| --- | --- |
| **Data collection** |  |
| Space group | P1 |
| Wavelength (Å) | 0.97852 |
| Unit cell dimensions |  |
| a,b,c (Å) | 53.993, 67.783, 101.456 |
| α,β,γ (°) | 96.644, 100.299, 89.938 |
| Resolution (Å) | 50.00-3.70 (3.83-3.70) |
| Observed reflections | 14847 |
| Completeness (%) | 98.5 |
| Redundancy | 3.5 (3.6) |
| Rmerge (%) | 19.6 (72.6) |
| Ⅰ/σ | 5.83 (1.88) |
| **Refinement** |  |
| Rwork/Rfree (%) | 23.87/30.16 |
| No. atoms |  |
| Protein | 8242 |
| Ligands | 166 |
| Water | 0 |
| B-factors |  |
| Protein | 63.08 |
| Ligands | 95.44 |
| Water | 0 |
| r.m.s. deviation |  |
| Bond lengths (Å) | 0.002 |
| Bond angles (°) | 0.560 |
| Ramachandran plot |  |
| Favoured (%) | 95.62 |
| Allowed (%) | 4.38 |
| Outliers (%) | 0.00 |

*Values in parentheses are for highest-resolution shell.

**Table S3. Residues contributed interaction between h4C1 and CD47.**

| **h4C1** | **CD 47** | **Contacts ^a^** | **Total** |
| --- | --- | --- | --- |
| **H chain** |  |  | 181 |
| T30 | T102 | 4 (1)^b^ |  |
| S31 | L101, T102 | 5, 8 (1) |  |
| Y32 | Q31, E35, L101, T102 | 1, 1, 5, 1 |  |
| N52 | T102 | 10 (1) |  |
| Y54 | Q1, T102, R103 | 6 (1), 7, 9 |  |
| N55 | T102 | 1 |  |
| E100 | FUC322 | 10 |  |
| G101 | T34, L101 | 2, 5 |  |
| H102 | T34, E35, V36, Y37,  D51, G52, A53, L101 | 5, 6, 9, 13,  6 (1), 2, 6, 7 |  |
| F103 | T99, L101, T102 | 3, 11, 12 |  |
| Y104 | Y37, E97, T99, | 12, 1, 5 (1) |  |
| W107 | A53, L54 | 4, 3 |  |
| Y110 | FUC322 | 1 |  |
|  |  |  |  |
| **L chain** |  |  | 36 |
| Y49 | L54 | 2 |  |
| Y50 | L54, K56 | 10, 6 (1) |  |
| R53 | L54, N55 | 17 (2), 1 |  |

a Numbers represent the number of atom-to-atom contacts between h4C1 and CD47 residues, which were analyzed by the Contact program in CCP4 suite (the distance cutoff is 4.5 Å).

b Numbers in the parentheses represent the number of hydrogen bonds between h4C1 and CD47 residues which were analyzed by the Contact program in CCP4 suite (the distance cutoff is 3.5 Å).

**Materials and methods**

***Plasmid construction and protein purification***

The human CD47-ECD (residues 1-117, with a C15G mutation) coding region was cloned into the pCAGGS vector (Addgene) with six histidines at the C terminus using the *Nsi*I and *Xho*I restriction sites. HEK 293T cells were transiently transfected by plasmid pCAGGS-CD47-ECD and then cultured in an incubator with 5% CO_2_ at 37°C for protein expression. The CD47-ECD protein was sequentially purified through a HisTrap excel^®^ column (GE Healthcare) and Superdex-200^®^ column (GE Healthcare) in a buffer containing 20 mM Tris and 150 mM NaCl (pH 8.0). The mutated CD47-ECD (N32A or N55A) and PD-L1-ECD proteins were produced with the same methods.

The H chain and L chain coding genes of h4C1 or BsAb were separately cloned into pCAGGS vectors and co-transfected into HEK 293T (ATCC) cells. The culture medium was changed to fresh DMEM (Gibco) without FBS 6 h after transfection. The supernatants were collected on the third day after transfection, and the proteins were purified with a HiTrap protein A HP^®^ column (GE Healthcare) and subsequently a Superdex-200^®^ column (GE Healthcare) in a buffer containing 20 mM phosphate and 150 mM NaCl (pH 7.0).

The h4C1 mAb proteins were digested with the Human IgG Fab and F (ab’)2 Preparation Kits (Thermo Scientific) according to the manufacturer’s instructions. The Fab fragment was purified by HiTrap Protein A HP^®^ column (GE Healthcare) and Superdex-200^®^ column (GE Healthcare) chromatography in a buffer containing 20 mM Tris (pH 8.0) and 150 mM NaCl.

***Flow cytometry of CD47/SIRPα blockage assays***

The activity of h4C1 to block CD47 and SIRPα binding was assessed by flow cytometry. HEK 293T cells were transiently transfected with the pEGFP-N1-SIRPα expression plasmid for 24 h. The CD47-ECD protein with six histidines at its C-terminus (5 μL at a concentration of 50 μg/mL) was mixed with antibody h4C1 or isotype IgG (95 μL at a concentration of 30 μg/mL) and incubated at 4°C for 1 h. Then, 100 μL of the mixture was added to 1 × 10^6^ HEK 293T cells expressing SIPRα and incubated at 4°C for another 60 min. After washing with PBS three times, the cells were stained with secondary PerCP-anti-his antibody (Abcam) for 30 min and analyzed using flow cytometry (BD FACSCalibur Flow Cytometer). The activity of BsAb to block CD47 /SIRPα and PD-1/PD-L1 binding was assessed by the same method.

***SPR***

SPR measurements were performed at room temperature using a BIAcoreT100^®^ system with CM5^®^ chips (GE Healthcare). For all measurements, a buffer consisting of 150 mM NaCl, 10 mM HEPES, pH 7.4, and 0.005% (v/v) Tween-20 was used as running buffer, and all proteins were exchanged into this buffer in advance through gel filtration. The blank channel of the chip served as the negative control. The h4C1 mAb or BsAb was immobilized on the chip with anti-human IgG at B70 response units. Gradient concentrations of WT CD47-ECD, CD47 mutants (from 6.25 nM to 0.39 nM with doubling dilution) or PD-L1-ECD (from 250 nM to 15.63 nM with doubling dilution) were then flowed over the chip surface. After each cycle, the sensor surface was regenerated with 3 M MgCl_2_. The affinity was calculated using a 1:1 (Langmuir) binding fit model with BIAevaluation^®^ software.

***Macrophage based phagocytosis assays***

*In vitro* phagocytosis assays were performed as previously described^1^. Briefly, the Raji cell line labeled with CSFE (Invitrogen) was used as target cells. Murine BMDMs from 6- to 8-week-old BALB/c mice were plated in 12-well plates and cultured with complete RPMI-1640 (Invitrogen) medium containing 20 ng/mL murine M-CSF (PeproTech). Before analysis, macrophages were changed to serum-free medium. Phagocytosis reactions were performed using 5 × 10^5^ macrophages and 2.5 × 10^6^ target cells. Cells were co-cultured for 4 h at 37°C in the presence of h4C1 (from 10 μg/mL to 0.16 ng/mL with serial dilutions) or isotype IgG. After co-culturing, cells were washed twice with PBS and scraped off from wells with a cell scraper. Finally, the cells were labeled with APC-anti-F4/80 (Sungene) for analysis by flow cytometry. The phagocytosis index was interpreted as the ratio of APC and CSFE double-positive cells in the total APC positive cells (phagocytosis macrophages/total macrophages), and the EC_50_ was calculated by fitting the phagocytosis index from serially diluted antibody to a sigmoidal dose-response curve. Statistical significance was calculated using Sidak correction for multiple comparisons in Prism 6^®^ (GraphPad).

***Tumor suppression evaluation in a lymphoma xenograft mouse model***

This study was approved by the Research Ethics Committee of the Institute of Microbiology, Chinese Academy of Sciences. All of the subjects provided written informed consent for the studies performed on their samples and publication of their cases. Animals used in this study (6- to 8-week-old male NCG mice) were bought from the Model Animal Research Center of Nanjing University. All mice were bred in pathogen-free conditions. The study was conducted in accordance with the principles of the Declaration of Helsinki and the standards of good clinical practice (as defined by the International Conference on Harmonization), and Chinese regulatory requirements, as stipulated by the Chinese Food and Drug Administration.

Human lymphoma xenografts were established in the NOD-Prkdc^em26Cd52^Il2rg^em26Cd22^/Nju (NCG) mouse model as previously described^2^. Briefly, 1 × 10^6^ luciferase-labeled Raji cells (Zhong Qiao Xin Zhou Biotechnology) were injected subcutaneously into the hind flank of 6- to 10-week-old NCG mice. Mice were monitored for engraftment via bioluminescent imaging from IVIS Spectrum (Perkin Elmer) 7 days after tumor cell incubation and assigned to three groups, with nine mice per group. Each mouse was daily administered with 200 μg h4C1, Hu5F9, or isotype control mAb for 2 weeks. During the experiment period, the mice were imaged once a week to obtain the bioluminescence images, and the signal intensity of the luciferase radiance and luciferase imaging analysis were determined as described previously^3^. Statistical significance was determined by Student’s *t* test using Prism 6^®^ (GraphPad).

***Crystal screening and structure determination***

The CD47-ECD protein and h4C1-Fab fragment were mixed at a molar ration of 1:1. The mixture was incubated on ice for 60 min and further purified over a Superdex-200^®^ column (GE Healthcare). CD47-ECD/h4C1 complexes at 5 and 10 mg/mL were used for crystal screening by the vapor-diffusion sitting-drop method at 18°C. Diffracting crystals were obtained at a concentration of 10 mg/mL with conditions consisting of 20% (w/v) polyethylene glycol 3350, 0.1 M Bis Tris propane (pH 6.5), and 0.2 M potassium thiocyanate (Morpheus MD1-29-2 kit, Molecular Dimensions).

Crystals were flash-cooled in liquid nitrogen with cryoprotectant containing precipitant buffer plus 30% glycerol. The diffraction data were collected at the Shanghai Synchrotron Radiation Facility (SSRF) BL19U, and all data were processed with HKL2000^4^. The complex structure was solved by molecular replacement with Phaser^5^ using models from the Protein Data Bank (PDB codes: 2JJS, 5O4G, and 5TH9). The structure was modeled by iterative cycles of manual building and refinement using Phenix^6^ and COOT^7^. The stereochemical qualities of the final model were assessed with MolProbity^8^. The protein interfaces were analyzed using COOT and PISA^9^. Data collection and refinement statistics are summarized in Table S2. All structural figures were generated using Pymol (<http://www.pymol.org>).

***Hemagglutination assay***

Healthy human blood samples were provided by volunteers. Samples were treated with anticoagulant agent, and mixed with human lymphocyte separation medium (TBD). After separation and washing two times (centrifugation at 2000 rpm for 20 min), RBCs were mixed with phosphate buffer to generate a 6% (v/v) cell suspension. Then, 50 μL RBCs were added to a 96-well round bottom plate and mixed with 50 μL various amounts (ranging from 2.00 mg/mL to 11.3 ng/mL) of antibodies. The plate was incubated at 37°C for 4 h. Hemagglutination was defined by the red flocculation in the supernatant, and a lack of a significant change was defined by the colorless supernatant and the sinking of whole RBCs.

**References**

1 Zhang, H. *et al.* HIF-1 regulates CD47 expression in breast cancer cells to promote evasion of phagocytosis and maintenance of cancer stem cells. *Proc. Natl Acad. Sci. USA* **112**, E6215-6223, (2015).

2 Piccione, E. C. *et al.* A bispecific antibody targeting CD47 and CD20 selectively binds and eliminates dual antigen expressing lymphoma cells. *MAbs* **7**, 946-956, (2015).

3 Kuchimaru, T. *et al.* A luciferin analogue generating near-infrared bioluminescence achieves highly sensitive deep-tissue imaging. *Nat. Commun.* **7**, 11856, (2016).

4 Minor, W., Cymborowski, M., Otwinowski, Z. & Chruszcz, M. HKL-3000: the integration of data reduction and structure solution--from diffraction images to an initial model in minutes. *Acta. Crystallogr. D. Biol. Crystallogr.* **62**, 859-866, (2006).

5 McCoy, A. J. *et al.* Phaser crystallographic software. *J. Appl. Crystallogr.* **40**, 658-674, (2007).

6 Terwilliger, T. C. *et al.* Iterative model building, structure refinement and density modification with the PHENIX AutoBuild wizard. *Acta. Crystallogr. D. Biol. Crystallogr.* **64**, 61-69, (2008).

7 Emsley, P., Lohkamp, B., Scott, W. G. & Cowtan, K. Features and development of Coot. *Acta. Crystallogr. D. Biol. Crystallogr.* **66**, 486-501, (2010).

8 Chen, V. B. *et al.* MolProbity: all-atom structure validation for macromolecular crystallography. *Acta. Crystallogr. D. Biol. Crystallogr.* **66**, 12-21, (2010).

9 Krissinel, E. & Henrick, K. Inference of macromolecular assemblies from crystalline state. *Journal of molecular biology* **372**, 774-797, (2007).
