# Supplementary material for: The identification of a CD47-blocking “hotspot” and design of a CD47/PD-L1 dual-specific antibody with limited hemagglutination
Source: Signal Transduct Target Ther. 2020 Mar 6;5:16. doi: 10.1038/s41392-020-0121-2 (PMC7058617; doi:10.1038/s41392-020-0121-2)
Supplement: Supplementary file 2 — Supplemental Material [file 41392_2020_121_MOESM2_ESM.pdf]

Preliminary Full wwPDB X-ray Structure Validation Report (i)

Mar 7, 2019 – 06:39 AM EST

This is a Preliminary Full wwPDB X-ray Structure Validation Report.

This report is produced by the standalone wwPDB validation server.
The structure in question has not been deposited to the wwPDB.
This report should not be submitted to journals.

We welcome your comments at *validation@mail.wwpdb.org*
A user guide is available at
https://www.wwpdb.org/validation/2017/XrayValidationReportHelp
with specific help available everywhere you see the (i) symbol.

PRELIMINARY VALIDATION REPORT

The following versions of software and data (see references (i)) were used in the production of this report:

MolProbity	:	4.02b-467
Mogul	:	1.7.3 (157068), CSD as539be (2018)
Xtriage (Phenix)	:	1.13
EDS	:	rb-20031633
Percentile statistics	:	20171227.v01 (using entries in the PDB archive December 27th 2017)
Refmac	:	5.8.0158
CCP4	:	7.0 (Gargrove)
Ideal geometry (proteins)	:	Engh & Huber (2001)
Ideal geometry (DNA, RNA)	:	Parkinson et al. (1996)
Validation Pipeline (wwPDB-VP)	:	rb-20031633

1 Overall quality at a glance (i)

The following experimental techniques were used to determine the structure:
X-RAY DIFFRACTION

The reported resolution of this entry is 3.68 Å.

Percentile scores (ranging between 0-100) for global validation metrics of the entry are shown in the following graphic. The table shows the number of entries on which the scores are based.

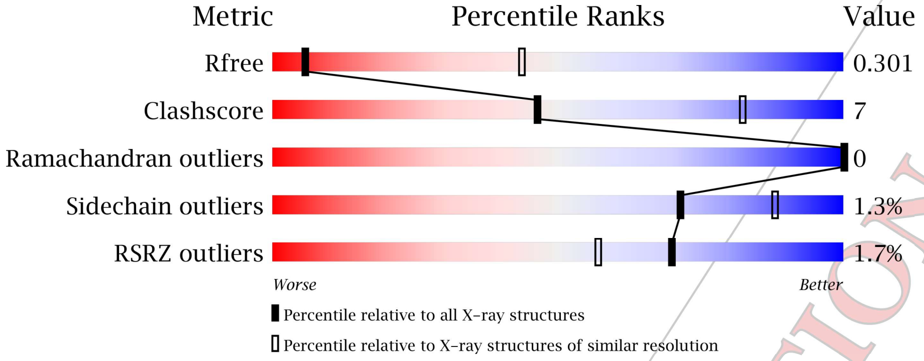

Metric	Whole archive (#Entries)	Similar resolution (#Entries, resolution range(Å))
R_{free}	111664	1320 (3.86-3.50)
Clashscore	122126	1424 (3.86-3.50)
Ramachandran outliers	120053	1374 (3.86-3.50)
Sidechain outliers	120020	1371 (3.86-3.50)
RSRZ outliers	108989	1214 (3.86-3.50)

The table below summarises the geometric issues observed across the polymeric chains and their fit to the electron density. The red, orange, yellow and green segments on the lower bar indicate the fraction of residues that contain outliers for >=3, 2, 1 and 0 types of geometric quality criteria. A grey segment represents the fraction of residues that are not modelled. The numeric value for each fraction is indicated below the corresponding segment, with a dot representing fractions <=5% The upper red bar (where present) indicates the fraction of residues that have poor fit to the electron density. The numeric value is given above the bar.

Mol	Chain	Length	Quality of chain
1	A	214	81% 18%
1	H	214	79% 21%
2	B	214	3% / 85% 15%
2	L	214	83% 16%
3	G	123	7% / 85% 14% •

Continued on next page...

PRELIMINARY VALIDATION REPORT

Continued from previous page...

Mol	Chain	Length	Quality of chain
4	E	122	2% 84% 16%

The following table lists non-polymeric compounds, carbohydrate monomers and non-standard residues in protein, DNA, RNA chains that are outliers for geometric or electron-density-fit criteria:

Mol	Type	Chain	Res	Chirality	Geometry	Clashes	Electron density
3	NAG	G	301	-	-	-	X
3	FUC	G	322	-	-	-	X
4	NAG	E	311	-	-	-	X
4	FUC	E	322	-	-	-	X

PRELIMINARY VALIDATION REPORT
